# Review of Colonic Anastomotic Leakage and Prevention Methods

**DOI:** 10.3390/jcm9124061

**Published:** 2020-12-16

**Authors:** Alex H. Fang, Wilson Chao, Melanie Ecker

**Affiliations:** 1 Texas Academy of Mathematics and Science, University of North Texas, Denton, TX 76203, USA; alexfang@my.unt.edu (A.H.F.); wilsonchao@my.unt.edu (W.C.); 2 Department of Biomedical Engineering, University of North Texas, Denton, TX 76203, USA

**Keywords:** anastomotic leakage, prevention, colorectal surgeries

## Abstract

Although surgeries involving anastomosis are relatively common, anastomotic leakages are potentially deadly complications of colorectal surgeries due to increased risk of morbidity and mortality. As a result of the potentially fatal effects of anastomotic leakages, a myriad of techniques and treatments have been developed to treat these unfortunate cases. In order to better understand the steps taken to treat this complication, we have created a composite review involving some of the current and best treatments for colonic anastomotic leakage that are available. The aim of this article is to present a background review of colonic anastomotic leakage, as well as current strategies to prevent and treat this condition, for a broader audience, including scientist, engineers, and especially biomedical engineers.

## 1. Introduction

Gastrointestinal surgery encompasses treatments for diseases of parts of the body involved in digestion, including the mouth, esophagus, stomach, small and large intestines, liver, pancreas, gallbladder, and anus. However, given that a large percentage of incidences of anastomotic leakage occurs in the area of the gastrointestinal tract involving the colon, this review focuses on colonic anastomotic leakage and treatments thereof [1]. Many of the following treatments, however, can also be applied to related regions of the system, such as the ileocolic and the rectum.

A colectomy, or a colon resection, is a surgical procedure to remove all or part of the large intestine to treat or prevent diseases and conditions that affect the colon. These conditions may include cancer, bowel obstructions, diverticulitis, and Crohn’s disease. In the past, open colectomy was considered to be the cornerstone operation, however, in recent years, less invasive laparoscopic colectomy has become more popular [2]. One of the deadliest complications of these surgeries is anastomotic leakage (AL), a major cause of postoperative mortality and morbidity. Following operations involving colonic resection, an artificial connection must be made through a procedure called anastomosis, which can lead to anastomotic dehiscence or AL, which has been reported in the literature to occur with varying rates depending on the type, technique, and site of surgery among others (Table 1). While, in historic studies, leak rates of up to 30% were reported [3], more recent studies have suggested rates under 3% [4]. Colonic anastomotic leak is defined as a “leak of luminal contents from a surgical join between two hollow viscera” [5]. In the case that the luminal contents were to leak out into the abdominal area, patients could experience fever, abscess, septicemia, metabolic disturbances, or multiple organ failure [5]. This can increase the need for reoperation, risk of local recurrence, increase morbidity and mortality, and can generally have a greater impact on the quality of life [6,7].

**Figure 1:**
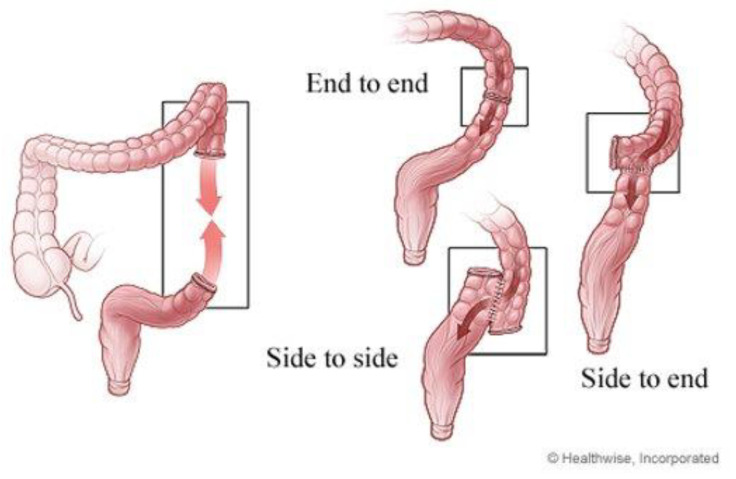
Types of anastomosis (with permission from ©Healthwise, Incorporated. www.healthwise.org).

AL has been a continuous problem in intestinal surgery for over a century. Various surgical techniques and prevention methods have been developed in the last few decades to contain these leakages [1]. Unfortunately, it seems that AL will continue to be a complication into the foreseeable future. There are several factors that describe an anastomosis. These factors include the orientation of the bowel, which dictates whether the anastomosis is side to side, end to end, or end to side (Figure 1); the technique used, which are handsewn and stapled; and the number the layers present, which is single or double layers [11].

Wound healing also plays a major role in a successful anastomosis. Therefore, AL is likely to occur when anastomotic healing is disrupted, even if the surgical procedure was conducted without flaws. It should be noted that wound healing in the gastrointestinal tract is different from that of cutaneous healing and is not yet fully understood [12]. The bowel wall of the colon consists of four layers, i.e., the mucosa, submucosa, muscularis propria, and serosa. Among these four layers, the submucosa, consisting mainly of collagen and elastin fibers, has historically been the most important layer in wound healing as it is the layer with the highest tensile strength. Within the first three to four days after gastrointestinal surgery, fibroblasts from the submucosa become active and start to deposit collagen [12]. After five days, the new tissue has already gained the strength and resilience of surrounding healthy tissue. After approximately four weeks post-op, the reorganization of collagen is almost finished, and the wound healing is about 90% complete. Therefore, the highest risk for AL is during the first few days after surgery for healthy patients [12,13,14]. However, the role of the other layers should not be neglected, since they are also essential during the wound healing process. The serosa seems to be important in providing a matrix for fibroblasts, while the interaction between bacteria, mucus and the mucosal layer also seem important to maintain homeostasis in which anastomotic healing can occur [12]. The formation of granulation tissue is also essential during normal wound healing, which includes fibrovascular tissue containing fibroblasts, collagen, and blood vessels [15]. Angiogenesis is crucial for the wound healing process because the wound needs to be supplied with oxygen, nutrients, and immune cells. Additionally, wastes must be removed from the injury site. New blood vessels and capillaries are usually formed within three days post injury and ensure sufficient tissue perfusion. Additionally, capillary growth is necessary to restore normal gut function, which includes the transport of nutrients from the mucosa into the bloodstream. There are multiple risk factors that can potentially affect wound healing, such as age, smoking and alcohol abuse, and even bacteria, such as *E. faecalis*, which has been shown to be associated with increased rates of AL and has been shown to possibly be contained by poly-phosphorylated polymer ABA-PEG20k-Pi20 in recent studies [16,17]. 

While most gastrointestinal surgeons and clinicians are probably aware of colonic anastomotic leakage and prevention methods thereof, this review aims to inform a broader scientific audience. When biomedical engineers become aware of these complications, they might be able to develop novel technologies in conjunction with clinicians to further mitigate the risk of AL and ultimately to improve patient outcomes. We have separated this review into the following four chapters: preoperative risk factors, intraoperative risk factors, postoperative management techniques, and emerging technologies.

## 2. Preoperative Risk Factors

Prevention and identification of risk factors, along with an early diagnosis of a colonic AL are crucial in the prevention of mortality. Patient factors are essential in the early diagnosis of AL. Even if the surgical operation is meticulous and finishes without flaw, if the patient’s ability to naturally heal is compromised or impaired, AL could still be a complication. Early diagnosis is also crucial for the prevention of mortality, with indications and symptoms including the presence of fever, oliguria, ileus, diarrhea, leukocytosis, and peritonitis [18]. Preoperative risk factors are generally divided into two types: modifiable, meaning that the patient can take measures to change them; or non-modifiable, meaning that they cannot be changed. 

### 2.1. Modifiable Risk Factors

Modifiable risk factors include alcohol, smoking, obesity, and medication among others [19].

#### 2.1.1. Alcohol and Smoking

Smoking and alcohol consumption exceeding 35 drinks per week are significantly associated with AL, regardless of patient age and surgical expertise [20,21,22,23]. Smoking, in one study, has been shown to increase the risk for AL by nearly four-fold to 17% as compared with nonsmokers at 5% [24]. Since the short-term cessation of smoking has not been shown to reduce leakage, recommendations are for patients to quit smoking four to eight weeks before operation and throughout the postoperative healing phase.

#### 2.1.2. Obesity and Body Mass Index (BMI)

Some studies have shown that obesity and a body mass index (BMI) greater than 25 can lead to an increased risk of AL [25]. Furthermore, the mortality rate after colectomy was 5% among obese patients as compared with 0.5% for non-obese patients [26]. While obesity has been linked to a higher risk of leakage, other measures, such as waist circumference and waist/hip ratios, can be more sensitive than BMI at predicting AL [27].

### 2.2. Non-Modifiable Risk Factors

Non-modifiable risk factors include gender, age, diabetes, tumor factors, to name just a few.

#### 2.2.1. Gender and Age

Males have a narrow pelvis, thus, increasing the technical difficulty of the surgery, Furthermore, from a study consisting of a total of 1349 patients, of which 754 were men, waist/hip ratio also seems to be correlated with higher rates of intraoperative complications, postoperative complications, and AL [27,28]. Studies have also shown that postoperative mortality rates due to AL increase after 60 years of age [29].

#### 2.2.2. Diabetes

While diabetes has not been shown to have a direct correlation with the presence of leakages, diabetic patients with leakage have had much higher mortality rates of 26.3% as compared with nondiabetic patients, who have a mortality rate of 4.5% [30].

## 3. Intraoperative Risk Factors

### 3.1. Surgical Techniques

Surgical techniques appear to have little to no statistical impact on the rate of the AL. The rate of leakage depends on a complex interplay between patient-related and procedure risk factors. In the past decade, laparoscopic and stapled anastomosis have risen for less invasive anastomosis. This raises the question of whether leak rates are comparable with more traditional surgical techniques such as open surgeries and sutures. In this section, we analyze and compare the anastomotic rate of stapled and hand-sewn anastomosis and laparoscopic and open anastomosis.

#### 3.1.1. Laparoscopic vs. Open Anastomosis

Surgeons can carry out anastomosis using laparoscopy or open laparotomy surgery. Laparoscopic surgery is a minimally invasive procedure in which the surgeon makes several small cuts into the abdominal wall to insert (1) a cannula to pass carbon dioxide into the abdominal cavity, (2) the laparoscope which is in essence a thin flexible tube with a light and camera at the end, and (3) surgical instruments. In contrast, conventional open surgery requires a large incision in the patient’s abdomen. The data regarding the effectiveness of laparoscopy reducing AL remain inconclusive. One Dutch group found that laparoscopies had increased rates of AL. However, this could be attributed to inexperience because many of the hospitals included in the study did not perform the required number of case volume to overcome the laparoscopic colectomy learning curve [31]. Most studies have concluded that laparoscopy offers a slight or no decrease in AL. In a study with 23,568 patients, 2.5% of laparoscopy led to AL in contrast to 4.5% of open surgeries, suggesting a statistically significant benefit to laparoscopy [32]. In contrast, a meta-analysis studying the effects laparoscopy has on mortality found that while laparoscopy decreased morbidity and mortality rates, it did not lessen the risk of AL [33]. The inconsistent and inconclusive results suggest that laparoscopy versus open surgery is not a driving factor in AL.

However, laparoscopic surgery has consistently been shown to have improved short-term and long-term outcomes as compared with conventional open surgery such as benefits in recovery time and length of hospital stay [34]. Other advantages of minimal access surgery such as less pain, lower narcotic requirements, a shorter period of ileus, shorter duration of disability, and a better cosmetic result have also been well documented. Since there is limited contact between the patient and surgeon during minimally invasive procedures, viral infections spreading between the two is also less likely [35]. However, laparoscopy is much more difficult to perform and may require extensive and highly specialized training. The use of minimally invasive techniques may also be limited since laparoscopic tools are not always suited to every surgery. The surgical site may be less accessible or larger tissues or tumors may need to be resected such as in rectal cancer surgery [36]. Additionally, laparoscopic surgery is generally slower and harder to perform than laparotomy because of the loss of tactile clues, but experienced surgeons in advanced laparoscopy can overcome these difficulties. Despite the difficulties with laparoscopic surgery, laparoscopic anastomotic surgery is preferable to open anastomosis because of the multitude of benefits of minimally invasive surgery.

#### 3.1.2. Stapled vs. Handsewn Anastomosis

**Figure 2:**
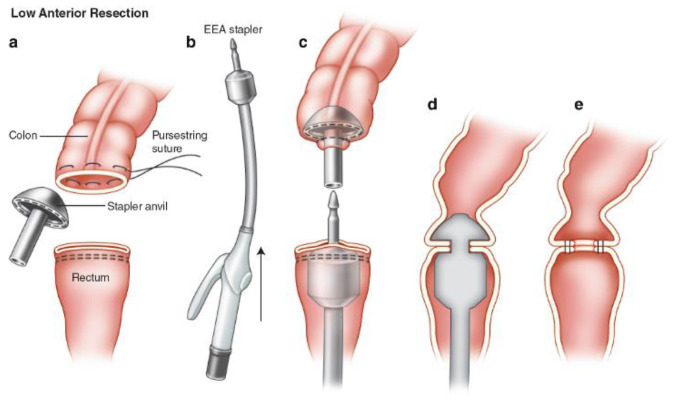
Schematic of an anastomosis performed with an end-to-end anastomosis (EEA) Stapler. (**a**) The stapler anvil is secured in the proximal colon with a purse string. (**b**) The EEA stapler is introduced through the anal canal and remaining rectum to the transverse rectal staple line. (**c**) The stapler spike is advanced slowly under close scrutiny of the abdominal operator and should be delivered near the midpoint of the transverse staple line. (**d**) The anvil is secured to the spike, and the stapler is closed under direct visualization. (**e**) The stapler is fired and then opened and removed per manufacturer’s instructions. Reprinted with permission from Springer International Publishing [38].

Surgical stapling was first pioneered by Hümér Hültl in 1908, but it did not gain popularity until much later because of the unreliability and cumbersomeness of early instruments. With modern advancements in the past 30 years, anastomosis stapling has become more reliable and widespread [37]. Currently, staples are generally used for end-to-end anastomosis while sutures are used for side-to-side anastomosis. The working principle of a circular end-to-end anastomosis (EEA) stapler can be seen in Figure 2 [38]. Staples can create a sturdy anastomosis in a relatively short amount of time and is much easier to master. However, because of its novelty as compared with handsewn anastomosis, it is much more expensive. More importantly, the data show that stapled anastomosis is only more effective situationally.

For an anastomosis to heal properly, three critical factors must be present, i.e., freedom from tension, adequate blood supply, and an inverted anastomosis. With stapling being the supposedly new and improved technology, there were hopes that it could decrease rates of anastomotic dehiscence and leakage. In a Cochrane Library review analyzing the effectiveness of staples in ileocolonic anastomosis from seven different randomized studies analyzing sutures versus staples, it was found that staples were more clinically effective in end-to-end ileocolic anastomosis, with staples having a 2.5% leakage rate as compared with hand-sewn anastomosis having a 6% leakage rate [39]. Other than this specific case, most studies have concluded that both techniques were effective and there was no statistically significant difference clinically between the two methods [39,40,41]. It has been shown that staples lead to less radiologic leaks then sutures, but most of these do not manifest clinically [3]. Though staples versus sutures may have a slight effect on the rate of anastomotic leak, it appears that other variables such as the location of anastomosis have a greater impact on the rate of leakage.

### 3.2. Compression Ring

**Figure 3:**
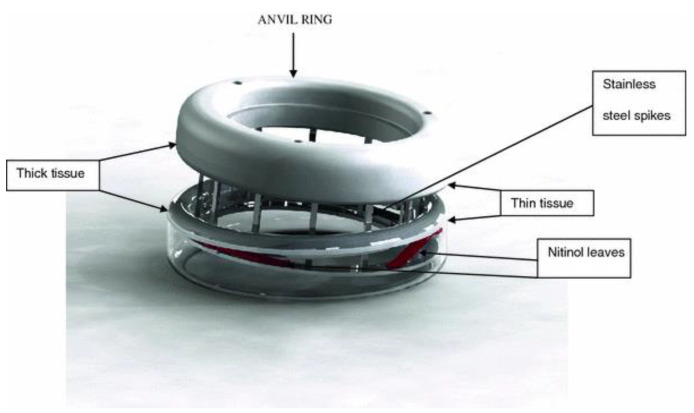
Illustration of the endoluminal compression anastomotic ring (EndoCAR), reprinted with permission from Springer [42].

The idea of a compression ring was first conceived in 1826. The idea was to compress two bowel walls together to cause a simultaneous necrosis and healing process leading to the joining of the two lumens. Over the years, several iterations have been made, however the most promising devices are the compression anastomotic clip (CAC) and the endoluminal compression anastomotic ring (EndoCAR, Figure 3) [42]. Previous iterations of anastomotic compression rings such as the Valtrac BAR and AKA-2 had several issues including the potential to increase anastomotic leak rate, bulkiness, and unreliability [43].

The CAC and EndoCAR both have shape memory and super-elastic properties making them more applicable in different thicknesses of tissue. Nitinol leaf springs, seen in Figure 3, maintain a continuous pressure at the anastomosis [44]. Animal trials were shown to have very good results, drastically reducing anastomotic leak rates. Reportedly, in animals, compression rings were able to decrease scarring as compared with stapling, and decreased inflammation [45]. Additionally, the use of compression rings has been reported to be safe in end-to-end anastomosis [46]. This shows that compression rings, especially the CAC and EndoCAR, are a promising technology and should be considered as an alternative to staples or sutures. Several compression rings, such as the NITI Compression Anastomosis Ring (CAR27) have been developed and FDA approved. It could be demonstrated that these devices are as effective as conventional circular staples [47].

### 3.3. Intraluminal Prosthesis (SBS Tube)

**Figure 4:**
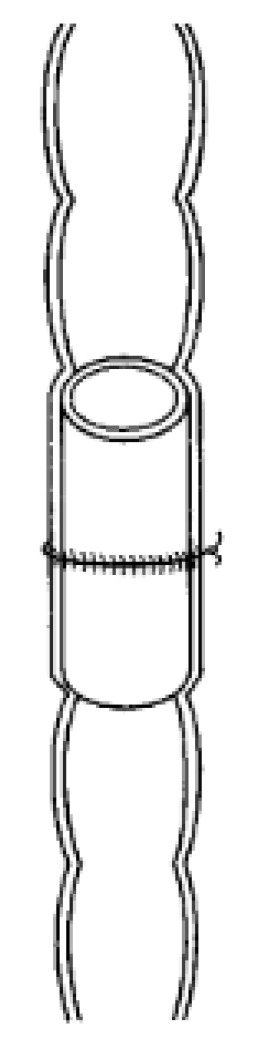
Schematic of an intraluminal prosthesis, called SBS tube. Reprinted with permission from Wiley [50].

The intraluminal prosthesis (SBS tube) (Figure 4) is a reabsorbable intraluminal prothesis given to reinforce the staple line, to allow for more adequate apposition, and to facilitate sutures [48,49]. The SBS tube supports the bowel during sewing by maintaining the luminal size and keeping the intestine in a fixed position assisting the surgeon. Afterwards, the tube dissolves almost immediately after the surgery. In a study conducted on pigs, the SBS tube was able to reduce suture tension and helped in precision apposition of the cut ends, which are both critical in anastomotic healing. Once the ends were side-by-side, the sticky tube surface helped maintain the position and made the sewing easy and precise [50]. The reduced suture tension as a result of the SBS tension also reduced anastomotic ischemia, which further improved healing. However, there were no differences in leakage test between the control and SBS tube-assisted surgery [50,51,52]. The SBS tube never gained widespread use because of the rise of laparoscopy, making it less applicable. Additionally, SBS tubes and other intraluminal devices have a small basis of evidence for their effectiveness in the literature, with most papers being either animal studies or small non-randomized human studies, and therefore clinicians are more reluctant to use them [53].

### 3.4. Coloshield

**Figure 5:**
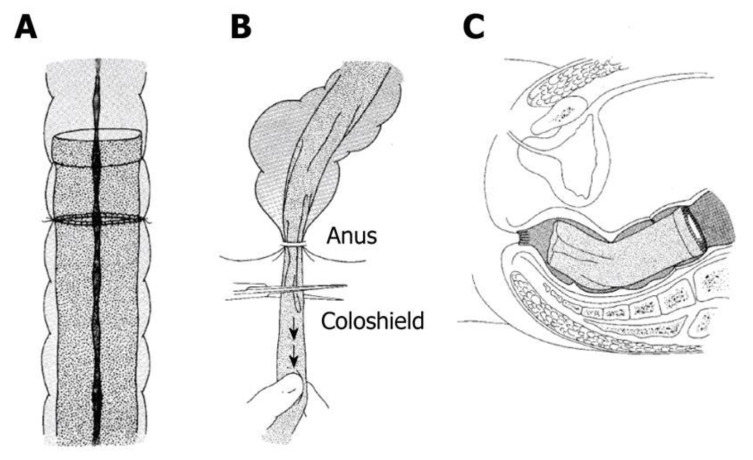
The coloshield, reprinted with permission from the Baishideng Publishing Group under CC BY-NC 4.0 [53]. (**A**) The coloshield is sutured to the submucosa of the bowel proximal of the anastomosis; (**B**,**C**) Slight traction is placed on the coloshield and it is cut so that it lies in the rectal ampulla.

The coloshield was developed in 1980 by Ravo and Ger and first tested on humans in 1984 [53]. This device is an endoluminal nonabsorbable silicon tube designed to be inserted during anastomosis (Figure 5) [54]. As seen in the Figure 5A, the coloshield is sutured to the submucosa of the bowel proximal of the anastomosis. Slight traction is placed on the coloshield and it is cut so that it lies in the rectal ampulla. In studies on dogs, it proved to be almost 100% effective and, in humans, 0–8.7% reported anastomosis-related complications [55]. Among these errors, a few were attributed to technical errors. Despite its promise, there is a dearth of large-scale randomized studies on this technology. Additionally, like other intraluminal devices, the rise of laparoscopy has made the coloshield more obsolete, and because of this, the coloshield never entered widespread use [53].

### 3.5. Mechanical Bowel Preparation (MBP)

Mechanical bowel preparation is oral preparation given prior to surgery to clear fecal material from the bowel lumen [56]. Since the first half of the 20th century, surgeons recognized that intestinal microbes play a role in AL. Patients were admitted preoperatively to decrease fecal load and to sterilize the bowel lumen through oral preparation. This led to postoperative infections decreasing by nearly 20% [57]. However, in the 1990s, as increasing use of antibiotics led to better outcomes of colonic surgery, mechanical bowel preparation started to be phased out. Numerous studies have found that mechanical bowel preparation was unnecessary and failed to reduce AL and other complications [56].

With the advent of electronic databases, more recent studies have found that combined with oral antibiotics, mechanical bowel preparation (MBP) can significantly reduce the chance of AL and surgical site infection [58,59]. Recently, in a study conducted at the University of Chicago, scientists found bacterium, such as *Enterococcus* faecalis, can drive anastomotic leak pathogenesis [58]. With new innovations, such as microbial metagenomic, and a better understanding of the intestinal microbiome, mechanical bowel preparation can more precisely target pathogens rather than broad-based MBP and mass destruction of the microbiome, as currently applied [60].

### 3.6. Intraoperative Air-Leak Testing

Intraoperative air-leak testing is commonly used to identify any risks of AL after colorectal surgery. The procedure is performed by insufflating 60 cc of air into the rectum through a syringe inserted into the anal canal, with the colon anastomosis under irrigation of saline. It is efficient in that it adds minimal time, risk, or cost to the procedure, while potentially being able to identify leaks in up to 25% of anastomosis [61,62]. However, it has been shown that colorectal AL rate did not significantly decrease in patients tested as compared with those who were not tested [63].

### 3.7. Splenic Flexure Mobilization

Mobilization of the splenic flexure is considered to be an essential step during laparoscopic anterior resection. If performed properly, it can achieve a tension-free anastomosis by providing sufficient colonic length, leading to a decrease in the risk of colorectal leakage [64]. This procedure is a crucial and essential part in all left-sided colorectal surgeries, allowing for an adequate resection with good blood supply. In the case that the operation takes place with the preservation of the left colic artery, it is argued that the need for the mobilization of the splenic fixture is unnecessary. However, it seems that the downside of performing a mobilization of the splenic flexure is that it can significantly increase operative time in exchange for a shorter length of stay [61].

### 3.8. Goal-Directed Fluid Therapy

Goal-directed therapy (GDT) is a term used to describe the use of cardiac output to perioperatively guide intravenous fluid and inotropic therapy. The goal is to optimize the balance between tissue oxygen supply and demand, which is done by balancing the patient’s fluid status between hypovolemia and hypervolemia. GDT has been shown to improve the postoperative outcome in patients undergoing high-risk surgery, with shorter hospital stays, faster gut function recovery, and overall less morbidity [65,66,67,68]. However, while goal-directed fluid therapy has been shown to reduce postoperative complications as well as postoperative morbidity and length of stay for major surgery, it has not been proven to reduce AL [19].

## 4. Postoperative Management Techniques

### 4.1. Severity Grading of Leakage

A method of detecting the severity of AL is through leakage score. Symptoms such as fever, increased heart, and respiratory rate, increased urinary production, and agitation or lethargy can be easily detected by the patient. Additionally, a local physical examination can detect signs of ileus, gastric retention, and fecal dehiscence, which can further measure the severity of AL [69]. After a leakage score exam, the clinician may perform a radiological examination to locate and detail the nature of the AL. The assessment of the severity of AL is important, as it determines the postoperative management thereof, some of which are outlined in the following subchapters. To date, there are different scoring and grading systems to either predict, diagnose, or grade the severity of AL [70]. The colon leakage score (CLS), for example, was developed to predict AL based on patient related and intraoperative risk factors [71]. In 2010, the International Study Group of Rectal Cancer proposed a definition and grading system for AL classifying AL into Grade A, B, and C. Grade A was defined as an asymptomatic leakage requiring no active therapeutic intervention [72]. Grade B was defined as a leakage that required active intervention without relaparotomy. Grade C was defined as a leakage that required relaparotomy. Grade A AL does not require any change in patient management. Grade B AL is managed through non-surgical intervention such as antibiotics and drainage. Grade C AL requires surgical intervention or the insertion of a stent. [73] Surgical intervention still remains critical in the management of Grade C anastomotic leakage goal to washout the colon and divert fecal matter [61].

### 4.2. Detection Techniques

Early diagnosis is critical to minimizing morbidity and mortality of AL. Currently, the most common methods for detecting AL are radiological techniques such as computerized tomography (CT) scan and water-soluble contrast enema (WSCE). However, the reliability of these methods depends on the location on the site of the anastomosis, the timing, and the expertise of clinician. WSCE has conflicting evidence of its effectiveness with some studies reporting 52.2% sensitivity and a false-positive rate of 6.4%, while others have reported an 80% success rate as compared with the 14% detection rate of CT scans [74]. This difference was further widened in patients with a distal anastomotic leak, proving that WSCE may be more reliable when diagnosing low AL. However, the CT scans have proven to be more detailed, which highlights the importance of an experienced radiologist.

In recent years, biomarkers such as MMP-2/9 and acute phase proteins, have gained attention and could develop into a promising and more accurate way to detect AL. In one study, through measuring white cell count, C-reactive protein, and procalcitonin, 95.4% of patients were correctly classified with a sensitivity of 90.9% and a specificity of 95.7% [75]. In another study with mice, MMP tracers were able to predict 71.4% of positive results and 66.6% of negative results [76]. However, the use of biomarkers will need further review and more rigorous testing.

### 4.3. Proximal Diverting Stoma

Proximal diversion is an operation to temporarily divert fecal matter to protect colonic anastomoses from pelvic sepsis or systemic illness. Although it does not prevent anastomotic leakage, it has been shown to mitigate the consequences of anastomotic leakage rate reducing the need for reoperation [77,78,79]. However, there are also several significant drawbacks to fecal diversion. Patients are subject to additional operations and may develop small bowel obstructions and acute kidney injury due to high stoma output, or a parastomal hernia. A study by Lightner et al. reviewed the role of temporary fecal diversion and concluded that diverting the stoma was significantly beneficial in patients undergoing low anterior resection, coloanal anastomosis, and ileal pouch-anal anastomosis. The authors also highlighted the importance of diverting stoma in immunosuppressed patients, since they are at the highest risk of anastomotic leakage. Despite many benefits arising from diverting fecal matter, it is very importance that the surgeon weighs the risks and advantages of constructing a stoma [79].

### 4.4. Draining

The evidence regarding the effectiveness of draining is inconsistent. In a study done by Zhao et al., trans anal draining proved to be promising with draining by reducing AL rates from 7.8% to 2.5% [80]. However, due to having very few cases with about 80 participants in each group and less than 10 people developing AL or bleeding, the difference was not statistically significant. In a meta-analysis done over eleven random controlled trials including 1803 patients, prophylactic drainage proved to be ineffective. However, some surgeons use drainage to guide exudation to flow out of the abdominal cavity to prevent anastomotic dehiscence. Nevertheless, only one of 20 clinical prophylactic drainage cases were effective in preventing and detecting AL and only lured surgeons into a false sense of security [81].

### 4.5. Antibiotics

Antibiotics are commonly used as the first line of treatment and can be used in combination with draining or reoperation. Treatments usually consist of a broad-spectrum antibiotic with Gram negative and anaerobic bacteria coverage [61]. Because of increasing multidrug resistant organisms, such as *Pseudomonas* and *Enterbacteriacea,* multidrug combination therapies are becoming increasingly necessitated [82,83]. In general, abscesses less than 3 cm in size can be managed with antibiotics alone if the patient is stable [84].

### 4.6. Exclusion of Perioperative Non-Steroidal Anti-Inflammatory Drugs (NSAIDS)

There is growing evidence that non-steroidal anti-inflammatory drugs (NSAIDs) should be used with caution in the postoperative period. A meta-analysis has demonstrated that non-selective NSAIDs were associated with an increased risk of AL. In recent years, a retrospective cohort study of over 13,000 bariatric and colorectal operations has shown a 24% increase in the risk of AL associated with NSAID use. This association is attributable to nonelective colorectal operations where the leak rate was 12.3% in the NSAID group and 8.3% in the non-NSAID group [19,85].

While some studies have shown that there was an association between increased leakage rates and NSAIDS, there are also studies that have concluded that there was a correlation [86]. Furthermore, according to a meta-analysis of NSAIDs and AL, the researchers found that the data from clinical findings were flawed and could be describing pre-existing bias [87]. However, there is still concern regarding NSAID usage and AL.

### 4.7. Stenting

In recent years, endoscopic self-expanding metal stents (SEMS) have become widely used for colorectal surgical complications with a reported success rate of around 80–85%, according to systematic reviews. Stents vary in their silicone coverage, from uncovered to partial coverage to full coverage, and in material, either metal or biodegradable. Despite their reported success, some complications may arise due to the use to SEMS to treat AL, including stent migration, perforation, and hemorrhages. While promising, the use of stents is still under review as migrations of the stents is a common problem throughout many studies and operations [88,89].

### 4.8. Vacuum Therapy

Vacuum-assisted wound closure (VAC) therapy or endoscopic placed negative pressure therapy has been shown to be a very promising in treating AL. It promotes healing of wounds by enhancing formation of granulation tissue, reducing oedema, increasing vascularity, and decreasing bacterial colonization [61,88]. In a study by Weidenhagen et al., 29 out of 34 patients with AL following resection reported successful treatments with VAC therapy [90]. Kuehn et al. also reported a success rate of 88% out of 41 patients [91].

## 5. Emerging Technologies

In recent years, novel technologies have been developed that are primarily aided in complementing the hand-sewn or stapled anastomosis through reinforcement.

### 5.1. Fibrin Glue

Fibrin glue consists of the following four components: fibrinogen, aprotinin, dried thrombin, and calcium chloride. Once prepared, the fibrin glue firmly adheres to the wound and sets into a rubber-like mass within seconds. It is applied to support the staple or suture line. The adhesive gains tensile strength over time, 70% of which is achieved after 10 minutes. As the wound heals, it slowly dissolves; aprotinin is added to slow the dissolution [92].

Fibrin glue has become the most novel option to treat anastomotic defects. It is often popularly used in obesity surgery. However, a randomized study by Carson et al. found its success with obese patients to be inconclusive with the control group having numerically more cases of anastomotic leak. Although there is inconclusive evidence supporting the use of fibrin glue, it has remained popular, due to a series of case studies, in which low leakage rates were reported [1,93]. In another study, where success was defined by the absence of further management interventions, success rates were achieved for 75% of colon and 16.7% of rectum surgeries. However, it was most effective in patients with minor cavities; 96.6% of patients had cavity size less than 0.5 × 1 cm achieving closure [88]. It can be concluded that the effectiveness of fibrin glue is largely dependent on the site and size of the AL.

### 5.2. Reinforcing the Staple Line

Several proposals have been made to reinforce the staple line, especially with bovine pericardium strips. Although several randomized studies have shown that reinforcing the staple line was safe, there have been no studies that showed that it directly decreased AL rate [13,94,95]. However, it has been shown that reinforcing the staple line can decrease anastomotic stricture [96].

Another way to reinforce the staple is through buttressing. Buttresses, or thin sheets made of different materials, are placed on one or both sides of the tissue to be stapled to provide additional support and apposition. Most of the evidence points to absorbable buttress materials providing a safer and effective control of preventing AL, but at an increased cost to non-reinforced lines [97]. Additionally, a study published by Mery et al. demonstrated that buttressing uniformly improved leak pressure and improved all types of staples [98]. This confirms that reinforcing or buttressing the staple line can be a promising intraoperative method of reducing anastomotic complication.

### 5.3. Polyphosphate Therapy to Suppress Bacterium that Cause anastomotic leakage (AL)

There is evidence that AL is caused by intestinal bacteria production of collagenase. Therefore, propositions to suppress bacterial collagenase production such as *Serratia marcescens* and *Pseudomonas aeruginosa* have been made. In a study with mice, Hyoju et al. effectively demonstrated that a polyphosphate (PPi-6) treatment was able to effectively reduce the colonization of collagenase producing bacterium and to reduce AL [99]. This could provide a non-invasive method to prevent the likelihood of AL.

### 5.4. Marine-Inspired Immunogenic Hydrogel Adhesive

**Figure 6:**
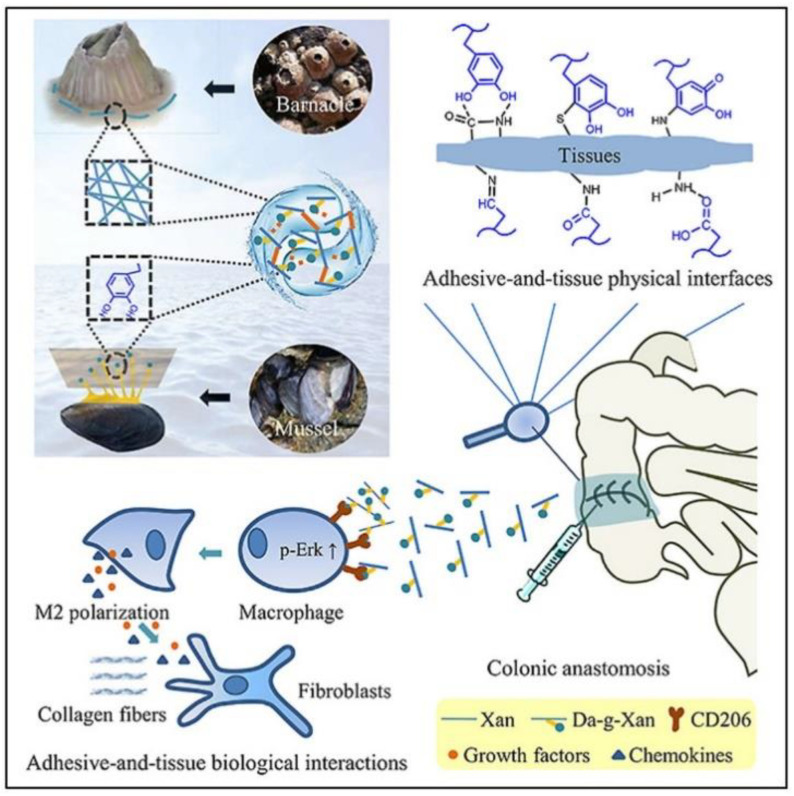
Background on the marine-inspired immunogenic hydrogel adhesive [100], reprinted with permission from science direct under CC BY-NC-ND 4.0.

Recently, a marine-inspired hydrogel adhesive was developed to prevent surgical AL. The hydrogel, dopamine-conjugated xanthan gum (Da-g-Xan), was based on a mussel’s adhesive, dopamine, the structure of barnacle cement proteins, and xanthan gum (Figure 6). The hydrogel adheres to wet tissue surfaces, like that of marine animals, thus improving bursting pressure. Additionally, intermolecular bonds allow the quick release of Da-g-Xan which can regulate inflammatory status and induce type 2 macrophage polarization [100]. However, this technique has only been tested on rats. Even so, the promising results warrant further testing to develop a new injectable and minimally invasive solution to AL.

## 6. Conclusions

Colonic AL remains a significant problem in colorectal surgery. Although developments have been made to manage AL, a frontrunner to treat and prevent AL has yet to rise. A combination of patient-specific risk analysis followed by carefully selected intraoperative and postoperative methods presents the best approach to manage AL. Preoperative and intraoperative therapies such as mechanical bowel preparation, intraoperative air-leak testing, splenic flexure mobilization, and goal-directed fluid therapy should be continued because they have been shown to reduce morbidity, even if they may not always prevent AL. Stents and vacuum therapy have been shown to be a promising way to treat AL, however, further research is recommended. Detection techniques such as CT scans and biomarkers should also be studied further to allow for early and reliable detection of AL. The success of emerging technologies, which mainly consist of topical sealants is tempered, likely because many of these treatments do not address wound healing and the vascularity of the tissue. Given the complexity of factors that influence the occurrence and severity of AL, further research is necessary that also includes the role of the intestinal microbiome and other factors on wound healing and successful anastomosis. Forthcoming engineering solutions should focus on mechanical aspects and also on wound healing. Clinicians and biomedical engineers should work together to develop the next generation of bioactive devices, similar to the marine-inspired hydrogel adhesive, to improve patient outcomes.

## Figures and Tables

**Table 1:** Reported rates of anastomotic leakage of different studies.

Reported Leakage Rate	Topic of Comparison or Study	Year Published	Reference
3.4–6%	Clinical colorectal surgery	2004	Chambers et al. [8]
1–30%	Anastomotic dehiscence	2009	Kingham et al. [3]
3–8%	Large bowel resection, colorectal cancer, left colon	2012	Oprescu et al. [9]
2.5–12%	Laparoscopic colorectal surgery	2010	Goriainov et al. [10]
2.7%	Population-based, retrospective cohort study	2017	Nikolian et al. [4]
